# Supplementary material for: Factors Predicting Post-Traumatic Positive and Negative Psychological Changes Experienced by Nurses during a Pandemic COVID-19: A Cross-Sectional Study
Source: Int J Environ Res Public Health. 2022 Jun 9;19(12):7073. doi: 10.3390/ijerph19127073 (PMC9223076; doi:10.3390/ijerph19127073)
Supplement: Supplementary file 1 [file ijerph-19-07073-s001.zip › ijerph-1717240-supplementary.pdf]

International Journal of
Environmental Research and Public Health

MDPI

Supplementary Materials

Factors Predicting Post-Traumatic Positive and Negative Psychological Changes Experienced by Nurses During a Pandemic COVID-19: A Cross-Sectional Study

Grzegorz Józef Nowicki, Barbara Ślusarska, Bożena Zboina, Aneta Jędrzejewska and Marzena Kotus

Table S1. Relationship between the positive outlook of the COVID-19 pandemic and selected sociodemographic and cognitive variables in woman.

Variables	Changes in Outlook Questionnaire – subscale Positive Change										
	Model A			Model B				Model C			
Sociodemographic variables:	b	SE	*p*	b	SE	*p*	R^2	b	SE	*p*	R^2
Age	0.004	0.02	0.84								
Place of residence (reference category: Urban area)	0	0	0	0	0	0		0	0	0	
Rular area	0.836	0.476	0.076	0.608	0.486	0.212		0.588	0.394	0.137	
Education (reference category: Bachelor`s degree)	0	0	0	0	0	0		0	0	0	
Master's degree or above	-0.106	0.456	0.86								
Postgraduate education (reference category: Specialist training course)	0	0	0	0	0	0		0	0	0	
Postgraduate diploma	-0.918	0.708	0.196								
Qualification course	-0.162	0.714	0.82								
Marital status (reference category: Married)	0	0	0	0	0	0		0	0	0	
Single	-0.986	0.5	0.05	-0.858	0.51	0.094		-0.385	0.415	0.354	
Divorced/Separated/Widowed	-1.514	0.751	0.045	-1.382	0.75	0.052		-0.183	0.619	0.768	
Living arrangements (reference category: Family)	0	0	0	0	0	0	3%	0	0	0	38%
Cohabitant/Flat mate or Roommate	-0.862	0.803	0.284								
Alone	-0.655	0.55	0.235								
Child(ren) in House (reference category: No)	0	0	0	0	0	0		0	0	0	
Yes	0.402	0.782	0.405								
Rotating shift schedule (reference category: No)	0	0	0	0	0	0		0	0	0	
Yes	0.598	0.483	0.217								
Have you nursed a patient diagnosed with COVID-19 (reference category: No)	0	0	0	0	0	0		0	0	0	
Yes	0.518	0.455	0.256								
Was there any training related to the coronavirus epidemic at work? (reference category: No)	0	0	0	0	0	0		0	0	0	
Yes	0.598	0.465	0.215								
Cognitive variables:											

IES-R – Total score	0.908	0.356	0.011	1.689	0.315	<0.001		1.629	0.319	<0.001
MSPSS – Total score	0.124	0.016	<0.001	0.059	0.016	<0.001		0.062	0.016	<0.001
SEQ – Sense of safety	1.677	0.276	<0.001	1.48	0.262	<0.001	37%	1.444	0.264	<0.001
SEQ – Reflection on safety	3.436	0.469	<0.001	1.37	0.453	0.003		1.353	0.452	0.003
MLQ – Total score	1.81	0.246	<0.001	1.226	0.237	<0.001		1.175	0.239	<0.001

Model A: univariable analysis; Model B: included significant factors in univariable analysis (performed separately for sociodemographic and cognitive factors); Model C: included all significant factors in univariable analysis; CIOQ: Changes in Outlook Questionnaire; IES-R: Impact Event Scale-Revised; MSPSS: Multidimensional Scale of Perceived Social Support; SEQ: Safety Experience Questionnaire; MLQ: Meaning in Life Questionnaire; b: standardised beta coefficient; SE: standard error

Table S2. Relationship between the negative perspective of the COVID-19 pandemic and selected sociodemographic and cognitive variables in woman.

Variables	Changes in Outlook Questionnaire – subscale Negative Change										
	Model A			Model B				Model C			
Sociodemographic variables:	b	SE	*p*	b	SE	*p*	R^2	b	SE	*p*	R^2
Age	-0.062	0.022	0.005	-0.016	0.033	0.624		-0.026	0.028	0.357	
Place of residence (reference category: Urban area)	0	0	0	0	0	0		0	0	0	
Rular area	0.548	0.527	0.299								
Education (reference category: Bachelor`s degree)	0	0	0	0	0	0		0	0	0	
Master's degree or above	-1.341	0.497	0.007	-1.234	0.491	0.012		-0.987	0.42	0.02	
Postgraduate education (reference category: Specialist training course)	0	0	0	0	0	0		0	0	0	
Postgraduate diploma	0.702	0.766	0.36	0.475	0.762	0.533		0.44	0.659	0.501	
Qualification course	2.021	0.525	<0.001	1.331	0.586	0.024		1.187	0.502	0.019	
Marital status (reference category: Married)	0	0	0	0	0	0		0	0	0	
Single	1.961	0.546	<0.001	2.085	0.961	0.03		1.187	0.502	0.026	
Divorced/Separated/Widowed	0.467	0.819	0.568	0.797	0.815	0.329	9%	1.118	0.705	0.114	
Living arrangements (reference category: Family)	0	0	0	0	0	0		0	0	0	38%
Cohabitant/ Flat mate or Roommate	0.624	0.607	0.307								
Alone	0.678	0.886	0.445								
Child(ren) in House (reference category: No)	0	0	0	0	0	0		0	0	0	
Yes	-1.266	0.526	0.017	1.051	0.959	0.274		0.857	0.822	0.298	
Rotating shift schedule (reference category: No)	0	0	0	0	0	0		0	0	0	
Yes	0.794	0.532	0.136								
Have you nursed a patient diagnosed with COVID-19 (reference category: No)	0	0	0	0	0	0		0	0	0	
Yes	0.391	0.501	0.436								
Was there any training related to the coronavirus epidemic at work? (reference category: No)	0	0	0	0	0	0		0	0	0	
Yes	-0.589	0.479	0.636								
Cognitive variables:											
IES-R – Total score	3.172	0.352	<0.001	2.594	0.374	<0.001	27%	2.624	0.363	<0.001	

MSPSS - Total	-0.055	0.019	0.004	-0.034	0.019	0.072	-0.042	0.018	0.021
SEQ – Sense of safety	-1.532	0.310	<0.001	-0.656	0.311	0.036	-0.606	0.301	0.045
SEQ – Reflection on safety	1.209	0.588	0.047	1.303	0.536	0.016	1.236	0.522	0.016
MLQ – Total score	-1.316	0.284	<0.001	-0.956	0.280	0.001	-0.784	0.273	0.004

Model A: univariable analysis; Model B: included significant factors in univariable analysis (performed separately for sociodemographic and cognitive factors); Model C: included all significant factors in univariable analysis; CIOQ: Changes in Outlook Questionnaire; IES-R: Impact Event Scale-Revised; MSPSS: Multidimensional Scale of Perceived Social Support; SEQ: Safety Experience Questionnaire; MLQ: Meaning in Life Questionnaire; b: standardised beta coefficient; SE: standard error
